# Prognostic value of smoking status in non-small-cell lung cancer patients treated with immune checkpoint inhibitors: a meta-analysis

**DOI:** 10.18632/oncotarget.18703

**Published:** 2017-06-28

**Authors:** Jung Han Kim, Hyeong Su Kim, Bum Jun Kim

**Affiliations:** ^1^ Division of Hemato-Oncology, Department of Internal Medicine, Kangnam Sacred-Heart Hospital, Hallym University Medical Center, Hallym University College of Medicine, Seoul 07441, Republic of Korea

**Keywords:** non-small-cell lung cancer, immune checkpoint inhibitor, smoking, meta-analysis

## Abstract

Immune checkpoint inhibitors (ICIs) have emerged as a new treatment option for patients with advanced non-small-cell lung cancer (NSCLC). Some studies with ICIs in NSCLC suggested that smoking history was associated with improved survival outcomes. We conducted this meta-analysis to investigate if survival benefits of ICIs in patients with advanced NSCLC are different according to smoking status. Electronic databases were searched for eligible studies. We included randomized controlled trials with the data of survival outcomes and extracted progression-free survival (PFS) or overall survival (OS) stratified by smoking status. From 6 studies, 2,389 ever-smokers and 413 never-smokers were included in the meta-analysis. In first-line treatment setting, ICIs tended to improve PFS in patients with smoking history (HR = 0.85 [95% CI, 0.71–1.10], *P* = 0.07). For never-smokers with advanced NSCLC, chemotherapy, not ICIs, was significantly associated with improvement of PFS (HR = 2.30 [95% CI, 1.23–4.28], *P* = 0.009). In more than second-line setting, ICIs significantly prolonged OS over that with chemotherapy in ever-smokers (HR = 0.70 [95% CI, 0.63–0.79], *P* < 0.00001). For never-smokers with NSCLC, however, ICIs failed to significantly improve OS (HR = 0.79 [95% CI, 0.59–1.06], *P* = 0.12). In conclusion, this meta-analysis indicates that ICIs can prolong survival over that with chemotherapy in ever-smokers with advanced NSCLC. However, ICIs failed to improve survival in never-smokers. These results suggest that smoking status may be a predictive marker for survival benefits to ICIs.

## INTRODUCTION

Lung cancer is the leading cause of cancer-related death all over the world [1, 2]. Tobacco smoking has been known as one of the predominant risk factors for lung cancer [3]. However, approximately 25% of lung cancer cases are not attributable to tobacco use [4]. Lung cancer patients with no history of smoking tends to has unique clinical characteristics, such as remarkable sex and geographic bias (female and Asian), higher incidence of adenocarcinoma, and higher rate of EGFR mutations [5, 6].

Immunotherapy has emerged as a new treatment option for patients with advanced non-small-cell lung cancer (NSCLC) [7]. The programmed death 1 (PD-1) receptors on activated T-cells are activated by the programmed death-ligand 1 (PD-L1) andPD-L2 expressed on tumor cells. The binding of PD-1 with PD-L1 and PD-L2 induces tumor immune escape by downregulating antitumoral T-cell function [8, 9]. Thus, inhibition of the PD-1/PD-L1 pathway can leads to anticancer immune responses. Immune checkpoint inhibitors (ICIs) refer to the anti-PD-1/PD-L1 monoclonal antibodies (mAbs) engineered to block PD-1/PD-L1-mediated inhibitory signals and restore antitumor immunity [10–16]. A number of randomized clinical trials in patients with advanced NSCLC have demonstrated that ICIs derived superior survival outcomes compared to chemotherapy [10–15]. ICIs have shown clinical benefits in cancer patients, but there is a great need to identify candidates who will respond to ICIs. Some studies showed the correlation between the efficacy of ICIs and PD-L1 expression on tumor cells and/or tumor-infiltrating immune cells [10, 11, 13]. As patients with no PD-L1 expression may also benefit from ICIs [14], however, the role of PD-L1 as a predictive marker is controversial. Tumor mutational load has been proposed as a possible marker for response to ICIs in NSCLC [17, 18]. High mutational frequency may be linked to the increase of neo-antigens recognized by T cells to mount effective anti-tumor T-cell responses [19]. Thus, tumor mutational burden may contribute to tumor immunogenicity, affecting tumor response to immunotherapy [17]. Smoking is associated with more mutational load [20], which may make tumors more immunogenic. Subgroup analysis of clinical trials with anti-PD-1 mAbs (nivolumab or pembrolizumab) in NSCLC suggested that smoking history was associated with improved survival outcomes [12, 15]. In the studies with an anti-PD-L1 mAb (atezolizumab), however, the overall survival benefit of ICIs over chemotherapy (docetaxel) was observed irrespective of smoking status [13, 14].

Therefore, it is unclear whether the efficacy of ICIs in patients with NSCLC is associated with smoking history. We conducted this meta-analysis of randomized controlled studies to investigate if survival benefits of ICIs in patients with advanced NSCLC are different according to smoking status.

## RESULTS

### Results of search

**Figure 1:**
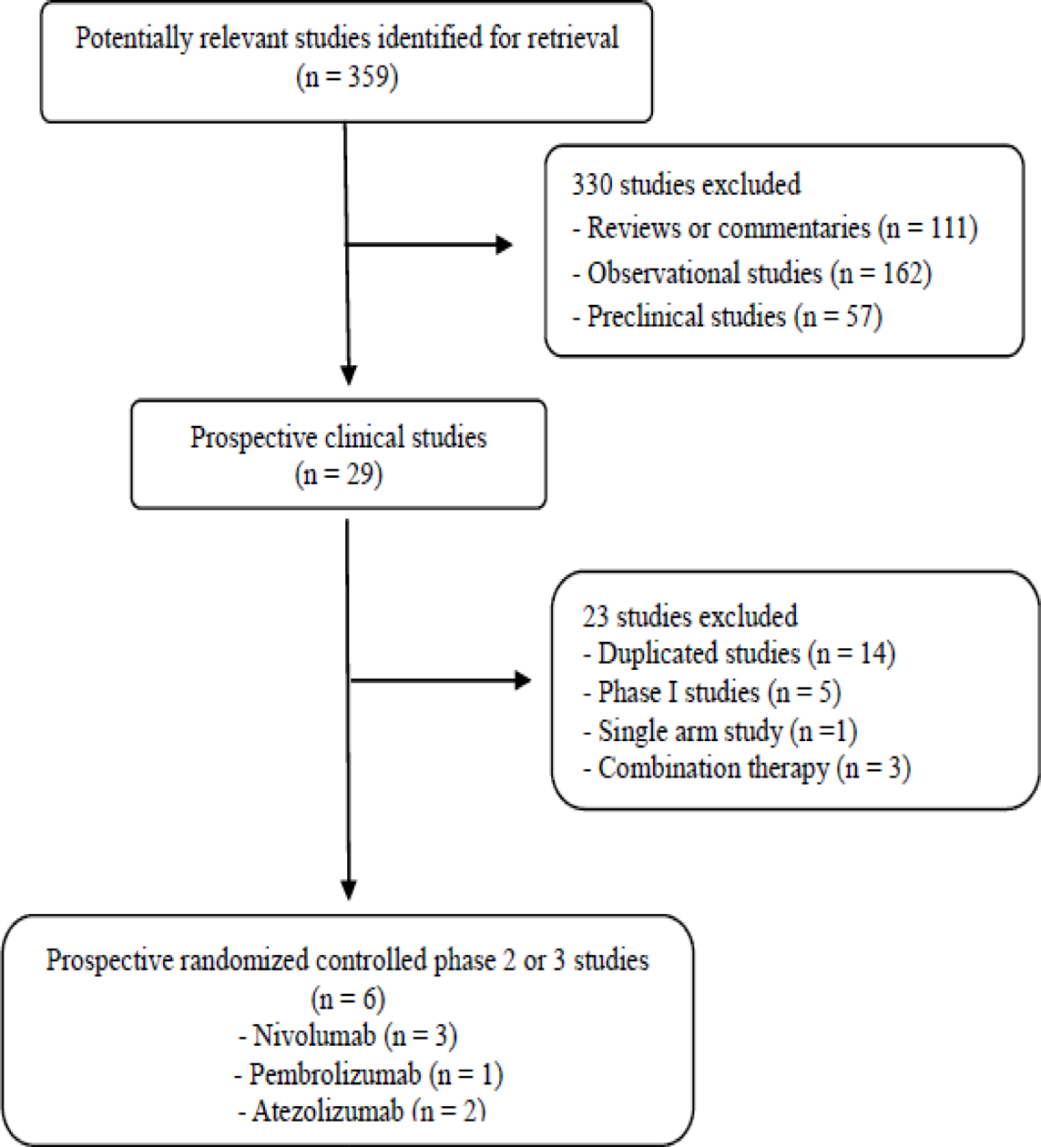
Flowchart of search process

Figure 1 shows the flowchart of studies through the selection process. A total of 359 studies were identified according to the searching strategy; 330 were excluded after screening the titles and abstracts. Out of the remaining 29 potentially relevant prospective studies, 23 were further excluded according to the inclusion criteria. Finally, six randomized phase 2 or 3 clinical trials were included in the meta-analysis [11–16].

### Characteristics of the included studies

Of the six studies, four were conducted in patients with previously treated NSCLC [11–14] and two were performed in first-line treatment setting [15, 16]. Five studies performed subgroup analysis according to smoking status (ever-smokers or never-smokers) [12–16]. In the remaining one study (CheckMate 017), subgroup analysis was available only in patients with a history of smoking [11]. Finally, the meta-analysis included 2,389 ever-smokers and 413 never-smokers.

Table 1 summarizes the characteristics and survival outcomes of the included studies. ICIs used in the studies included two anti-PD-1 mAbs (nivolumab and pembrolizumab) and one anti-PD-L1 mAb (atezolizumab).

**Table 1:** Summary of the six included studies

Author, study name (year)	Phase	Setting	PD-L1 cut-off	Treatments	Smoking status	No. of patients	HR for PFS (95% CI)	HR for OS (95% CI)
Brahmer *et al*., CheckMate-017 (2015)	3	2nd-line	Any	Nivolumab 3 mg/kg q2weeks vs. docetaxel	Ever Never	250 17	NA NA	0.59 (0.44–0.80) NA
Borghaei *et al*., CheckMate-057 (2015)	3	2nd-line	Any	Nivolumab 3 mg/kg q2weeks vs. docetaxel	Ever Never	458 118	NA NA	0.70 (0.56–0.86) 1.02 (0.64–1.61)
Fehrenbacher *et al*., POPLAR (2016)	2	2nd or 3rd-line	Any	Atezolizumab 1200 mg q3weeks vs. docetaxel	Ever Never	231 56	NA NA	0.75 (0.54–1.04) 0.55 (0.24–1.25)
Rittmeyer *et al*., OAK (2016)	3	2nd or 3rd line	Any	Atezolizumab 1200 mg q3weeks vs. docetaxel	Ever Never	694 156	NA NA	0.74 (0.61–0.88) 0.71 (0.47–1.08)
Socinski *et al*., CheckMate-026 (2016)	3	1st-line	≥ 1%	Nivolumab 3 mg/kg q2weeks vs. chemotherapy	Current Former Never	107 386 59	1.03 (0.66–1.62) 1.14 (0.89–1.47) 2.51 (1.31–4.83)	1.05(0.63–1.74) 1.09 (0.84–1.42) 1.02 (0.54–1.93)
Reck *et al*., KEYNOTE-024 (2016)	3	1st-line	≥ 50%	Pembrolizumab 200 mg q3weeks vs. platinum-based chemotherapy	Current Former Never	65 216 24	1.03 (0.66–1.62) 1.14 (0.89–1.47) 2.52 (1.31–4.83)	NA NA NA

PD-L1, programmed death-ligand 1; HR, hazard ratio; PFS, progression-free survival; OS, overall survival; CI, confidence interval; NA, not available.

### Progression-free survival in first-line treatment

**Figure 2:**
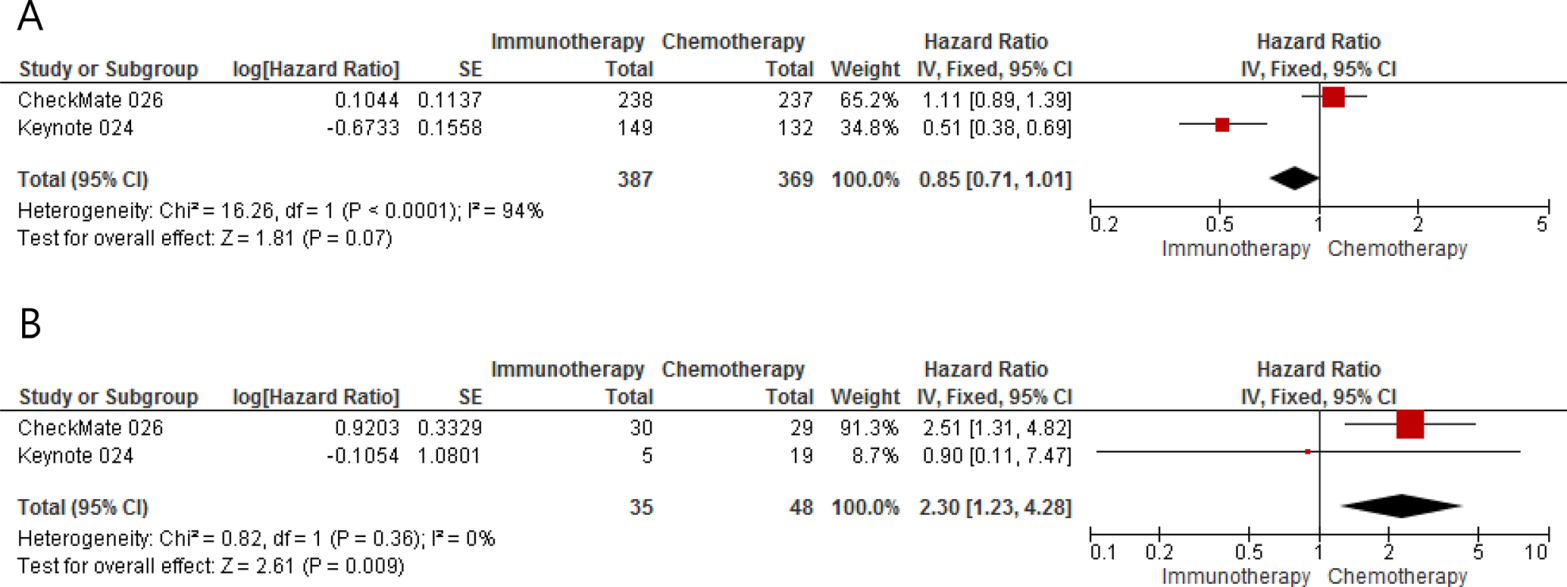
Forest plots of hazard ratios for progression-free survival in (**A**) ever-smokers and (**B**) never-smokers.

From 2 studies conducted in first-line setting, 756 current or former smokers with advanced NSCLC and 83 patients with no history of tobacco use were included in the meta-analysis of hazard ratios (HRs) and 95% confidence intervals (CIs) for progression-free survival (PFS) [15, 16]. Compared with chemotherapy, ICIs tended to improve PFS in patients with smoking history (HR = 0.85 [95% CI, 0.71–1.10], *P* = 0.07) (Figure 2A). The random-effect model was selected because there was a significant heterogeneity (*X*^2^ = 16.26, *P* < 0.0001, *I^2^* = 94%). For never-smokers with advanced NSCLC, chemotherapy, not ICIs, was significantly associated with improvement of PFS (HR = 2.30 [95% CI, 1.23–4.28], *P* = 0.009) (Figure 2B). There was no significant heterogeneity (*X*^2^ = 0.82, *P* = 0.36, *I^2^* = 0%).

### Overall survival in more than second-line treatment

**Figure 3:**
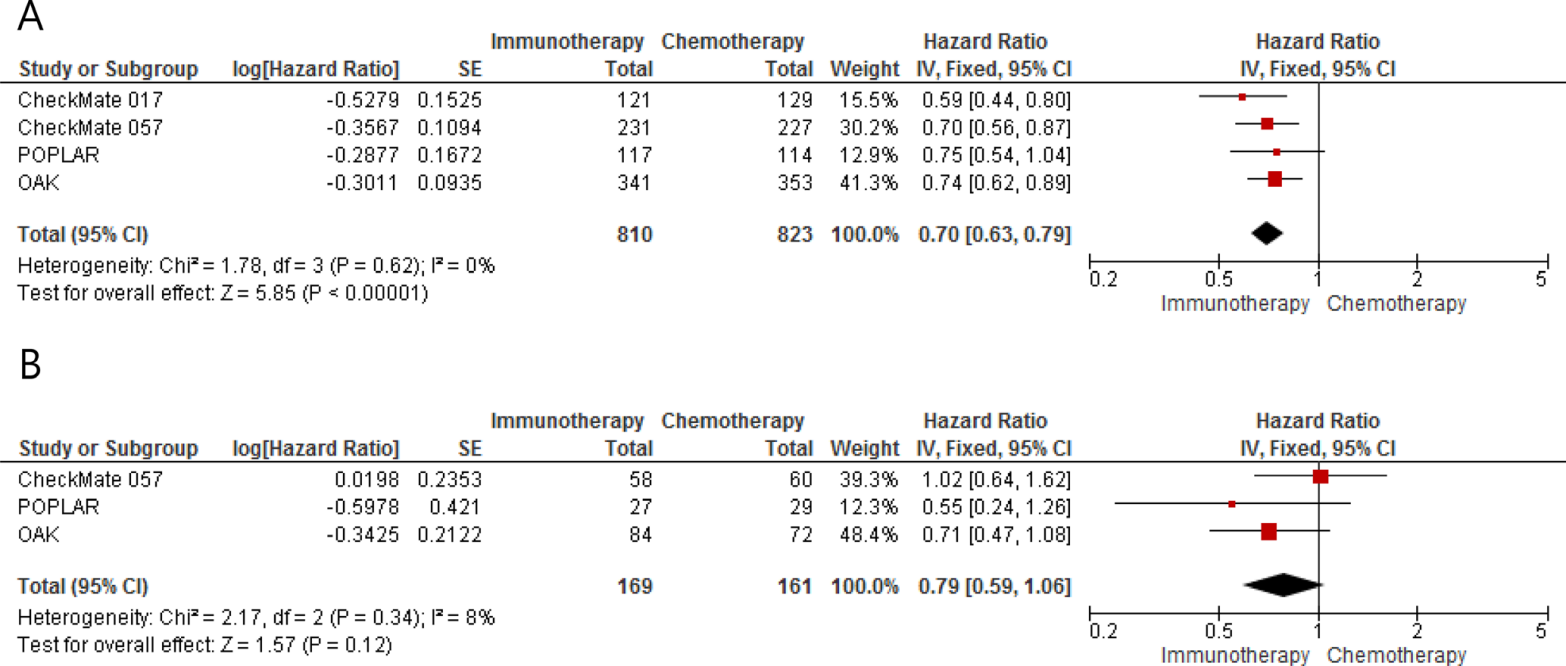
Forest plots of hazard ratios for overall survival in (**A**) ever-smokers and (**B**) never-smokers.

From 4 studies performed in patients with previously treated NSCLC [11–14], 1,633 ever-smokers and 330 never-smokers were included in the meta-analyses of HRs and 95% CIs for overall survival (OS). After the meta-analysis, we found that ICIs induced 30 % reduction of the death risk in ever-smokers with advanced NSCLC (HR = 0.70 [95% CI, 0.63–0.79], *P* < 0.00001) (Figure 3A). There was no significant heterogeneity (*X*^2^ = 1.78, *P* = 0.62, *I^2^* = 0%). For never-smokers with advanced NSCLC, ICIs failed to significantly improve OS, compared with chemotherapy (HR = 0.79 [95% CI, 0.59–1.06], *P* = 0.12) (Figure 3B). There was no significant heterogeneity (*X*^2^ = 2.17, *P* = 0.34, *I^2^* = 8%).

## DISCUSSION

In this meta-analysis, we investigated whether survival benefits of ICIs in advanced NSCLC were different between ever-smokers and never-smokers. We found that ICIs as a first-line or salvage therapy for advanced NSCLC could prolong PFS or OS over those with chemotherapy in ever-smokers, but not in never-smokers.

ICIs have proved survival benefit in patients with advanced NSCLC, but there is a critical need to identify predictive biomarkers associated with advantages from ICIs. Although some studies have showed the significant correlation between the efficacy of ICIs and PD-L1 expression level [10, 11, 13], the predictive value of PD-L1 expression is still controversial [14]. It is well known that various carcinogens in tobacco smoke are responsible for much of the mutagenesis in lung cancer. Smoking is linked to the expression of neoantigens and increased numbers of somatic mutations [17]. Thus, lung cancers in tobacco users show a higher mutational burden than those developing in never-smokers. Considering the findings that cancer types with a relatively high mutational burden tended to show better outcomes to ICIs [17, 18], mutational landscape of a given tumor may be an important predictive marker of response to ICIs [21]. Rizvi *et al*. recently reported the results of an interesting study to assess the effects of smoking on the mutational landscape and pembrolizumab response in NSCLC [17]. According to the molecular signature of smoking (frequency of C→A transversions in lung cancer exomes), they defined tumor samples as “transversion high (TH, ever-smoking signature)” or “transversion low (TL, never-smoking signature).” Patients with TH molecular signature had higher mutational burden and showed better clinical benefits with pembrolizumab. This result suggests that smoking status might be a predictive marker for clinical benefits to ICIs.

In this meta-analysis, ever-smokers with advanced NSCLC derived significant OS benefit from ICIs over chemotherapy (docetaxel) as a salvage therapy. ICIs also tended to prolong PFS for patients with a history of smoking in first-line treatment setting (*P* = 0.07). In never-smokers with NSCLC, however, ICIs failed to significantly improve survival (PFS or OS) regardless of treatment setting. These results indicate that smoking status is a simple but useful clinical predictive marker for survival benefits to ICIS in patients with advanced NSCLC. Interestingly, smoking status has also shown a significant clinical impact in NSCLC patients with epidermal growth factor receptor (EGFR) mutations [22, 23]. A meta-analysis by Zhang *et al*. reported that smoking history was detrimental to patients with NSCLC harboring EGFR mutations [22]. Among EGFR mutant NSCLC patients receiving EGFR tyrosine kinase inhibitors (TKIs), ever-smokers showed significantly shorter PFS than never-smokers. In a meta-analysis of randomized trials by Hasegawa *et al*., PFS benefit of EGFR-TKIs over platinum doublet chemotherapy was significantly better in patients with no smoking history [23]. The higher mutational burden in tobacco users might contribute to these results.

Of note, our study has several potential limitations. First, this meta-analysis included a limited number of studies. In first-line setting, especially, only two studies were available. Second, the current study included heterogeneous patients with various levels of PD-L1 expression. Smoking status might differently affect clinical outcomes of ICIs according to PD-L1 status. Last, this meta-analysis could not include patients who had received ICIs in combination with chemotherapy for first-line treatment of advanced NSCLC because there were no eligible studies in the literature [24, 25]. In a multi-cohort phase 1 study (CheckMate 012), however, a trend toward higher overall response rate and longer PFS was noted for patients with a history of smoking [25].

In conclusion, this meta-analysis indicates that ICIs, compared to chemotherapy, can prolong survival in ever-smokers with advanced NSCLC. However, ICIs failed to improve survival in never-smokers. These results suggest that smoking status might be a predictive marker for survival benefits to ICIs. Since this meta-analysis included heterogeneous clinical trials with a small number of never-smokers, further studies are still needed to evaluate the impact of smoking status on the survival benefits of ICIs in patients with advanced NSCLC.

## MATERIALS AND METHODS

### Searching strategy

The following terms were used for searching: ‘immune checkpoint inhibitor’, ‘nivolumab or pembrolizumab or atezolizumab or ipilimumab’, ‘advanced or metastatic’, ‘non-small-cell lung cancer or NSCLC’. We carried out a systematic search of electronic databases, such as PubMed, MEDLINE, EMBASE, and Google Scholar. In addition, we reviewed abstracts presented in the ESMO 2016 Congress or IASLC 17th WCLC. We retrieved all eligible studies and checked their bibliographies for other relevant articles. We also looked into all the references of identified relevant articles and reviews. When the data were unclear or incomplete, the corresponding authors were contacted to clarify data extraction.

### Inclusion criteria

Eligible studies were required to meet the following inclusion criteria: randomized controlled trials in advanced NSCLC; randomization of patients to either immunotherapy with ICIs or chemotherapy; performing subgroup comparison of PFS or OS by smoking status (ever-smokers or never-smokers); providing HRs with their 95% CIs for PFS or OS.

### Data extraction

The following data were collected from the eligible studies: the first author's name, year of publication, study phase, number of patients, treatment setting and regimen, PD-L1 expression level, PFS and OS stratified by smoking status and HRs with their 95% CIs.

Data extractions were carried out independently by two authors (BJK and HSK). If the two authors could not reach a consensus, the other (JHK) was consulted to resolve the dispute.

### Statistical analyses

Statistical values used in the meta-analysis were obtained directly from the original articles or abstracts. The effect size of PFS and OS was pooled through HR and its 95% CI, whereas the effect size of the other outcomes was evaluated via the number of patients. The heterogeneity across studies was examined by *Q* statistic and the *I*^2^ statistic. The fixed-effect model (Mantel–Haenszel method) was selected for pooling the homogeneous outcomes when *P* ≥ 0.1 and *I^2^* ≤ 50%, and the random-effects model (DerSimonian–Laird method) was applied for pooling heterogeneous outcomes when *P* < 0.1 and *I^2^* > 50%.

The plots show a summary estimate of the results from all the studies combined. The size of the squares shows the estimate from each study and reflects the statistical ‘weight’ of that study (the relative contribution of that study to the summary estimate). Results are graphically presented as forest plots with diamonds representing estimate of the pooled effect and the width of diamond representing its precision. The line of no effect is number one for binary outcomes, which, if not crossed by the diamond, indicates statistical significance. All *P*-values were from two-sided versions of the respective test and *P* < 0.05 was considered statistically significant. RevMan version 5.2 software was used to report outcomes.
